# Supplementary material for: Vangl2 promotes the formation of long cytonemes to enable distant Wnt/β-catenin signaling
Source: Nat Commun. 2021 Apr 6;12:2058. doi: 10.1038/s41467-021-22393-9 (PMC8024337; doi:10.1038/s41467-021-22393-9)
Supplement: Supplementary file 1 — Supplementary Information [file 41467_2021_22393_MOESM1_ESM.pdf]

Vangl2 promotes the formation of long cytonemes to enable distant Wnt/β-catenin signalling

Lucy Brunt, Gediminas Greicius, Sally Rogers , Benjamin D Evans, David M Virshup, Kyle CA Wedgwood, Steffen Scholpp

Supplementary Data Figures with Figure Legends

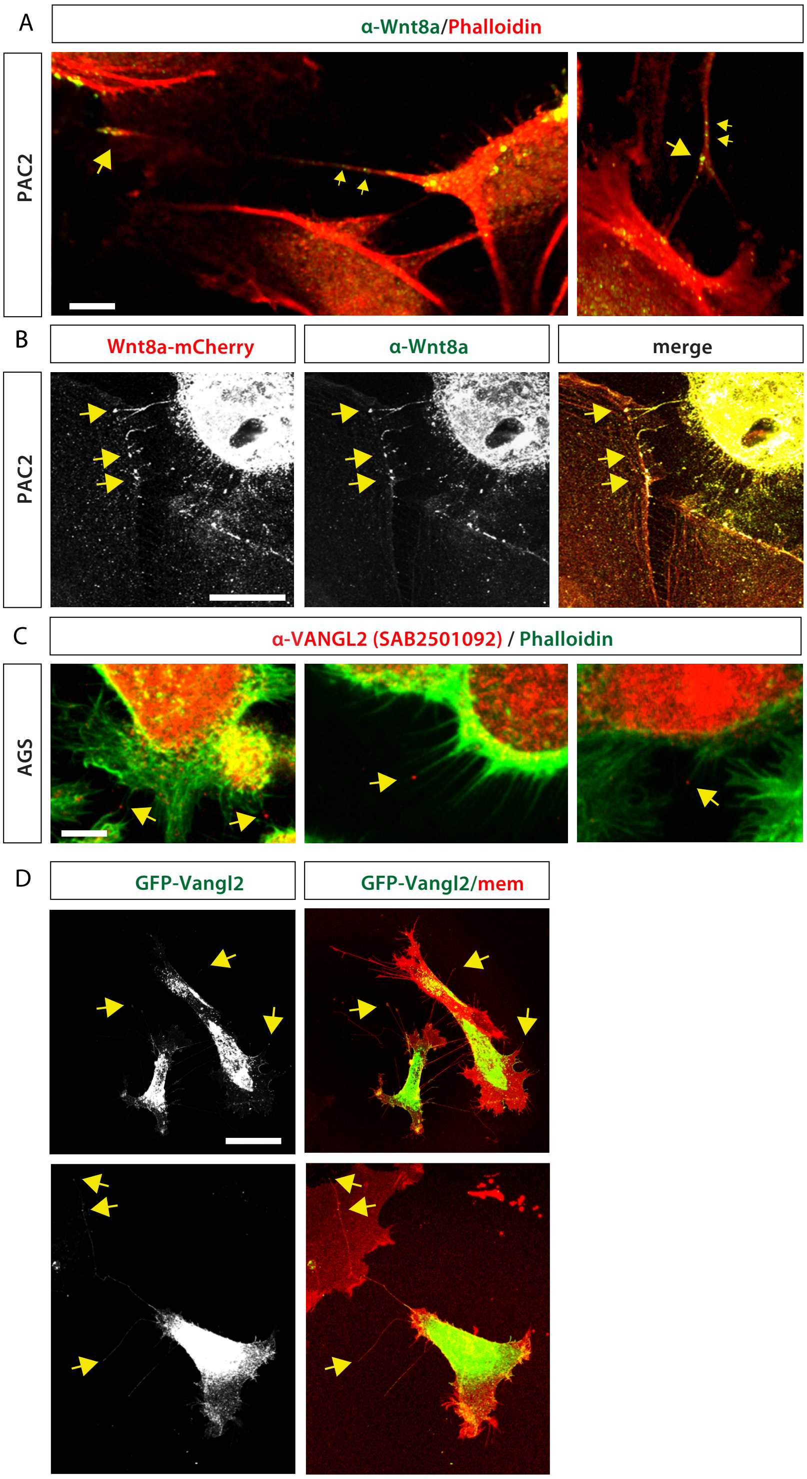

Supplementary Figure 1: Endogenous Wnt8a is localised at cytoneme tips. (A): PAC2 cells immunostained for anti-Wnt8a with FITC-labelled secondary antibody and counterstained with Phalloidin-TRITC. Yellow arrows mark endogenous Wnt8a expression on cytoneme tips and cytoneme protrusions. (n=5 cells). Scale bar= 5µm. (B): PAC2 cells transfected with Wnt8a-mCherry and immunostained with anti-Wnt8a with FITC-labelled secondary antibody. Panels show red (Wnt8a-mCh), green (anti-Wnt8a) and merge channels. Yellow arrows show co-localisation of transfected Wnt8a-mCh with anti-Wnt8a antibody on cytoneme tips contacting recipient cells. (n=5 cells). Scale bar= 5µm. (C): Endogenous Vangl2 is localised at cytoneme tips (yellow arrows). AGS (human gastric cancer cells) immunostained for anti-Vangl2 with AlexaFluor-647 secondary antibody and counterstained with Phalloidin-FITC. (n=5 cells). Scale bar= 5µm. (D) PAC2 cells transfected with mem-mCh and GFP-Vangl2. Yellow arrows show Vangl2 accumulations on cytoneme tips. Panels show merge and green (Vangl2) channel. n= 26 cells. Scale bar= 10µm.

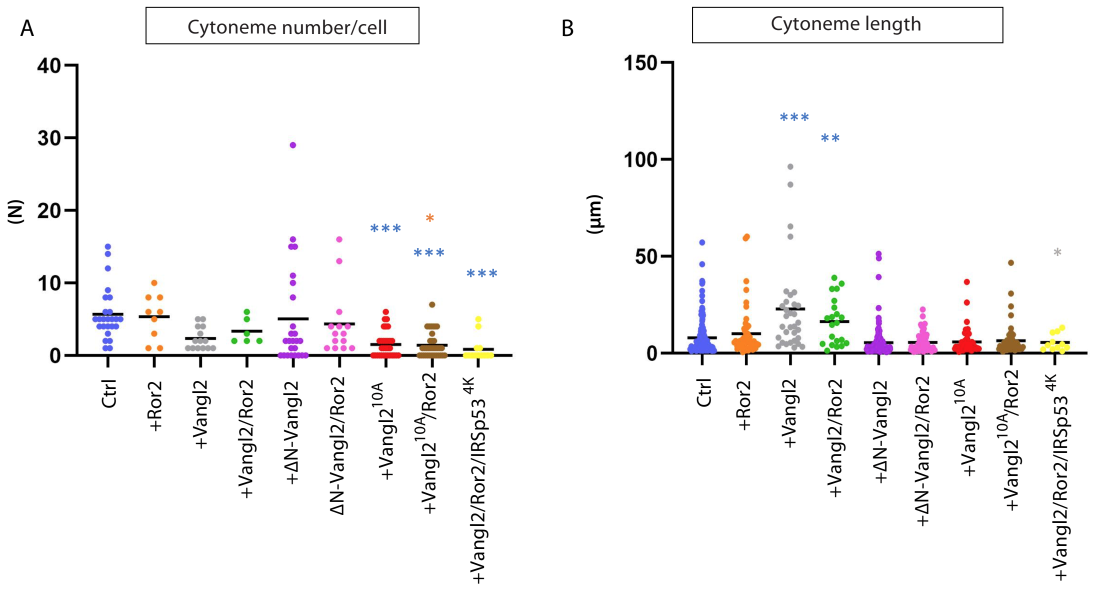

Supplementary Figure 2: Wnt8a-positive cytoneme number and length in PAC2 fibroblasts. Representations of cytoneme data from Figure 2 K, L as dot plots. (A): Number of cytonemes per cell (n per condition= 25, 9, 14, 6, 25, 14, 31, 36, 13 cells). (B): Length of Wnt8a positive cytonemes in PAC2 cells (µm). (n per condition= 139, 52, 32, 21, 131, 65, 47, 51, 11 cytonemes). Statistical significance: * ≤ 0.05, ** ≤ 0.01, *** ≤ 0.001. Blue stars significant to control, orange stars significant to Ror2, grey stars to Vangl2. Two-sided Kruskal-Wallis tests with Bonferroni correction for multiple tests. Source data are provided as a Source Data file.

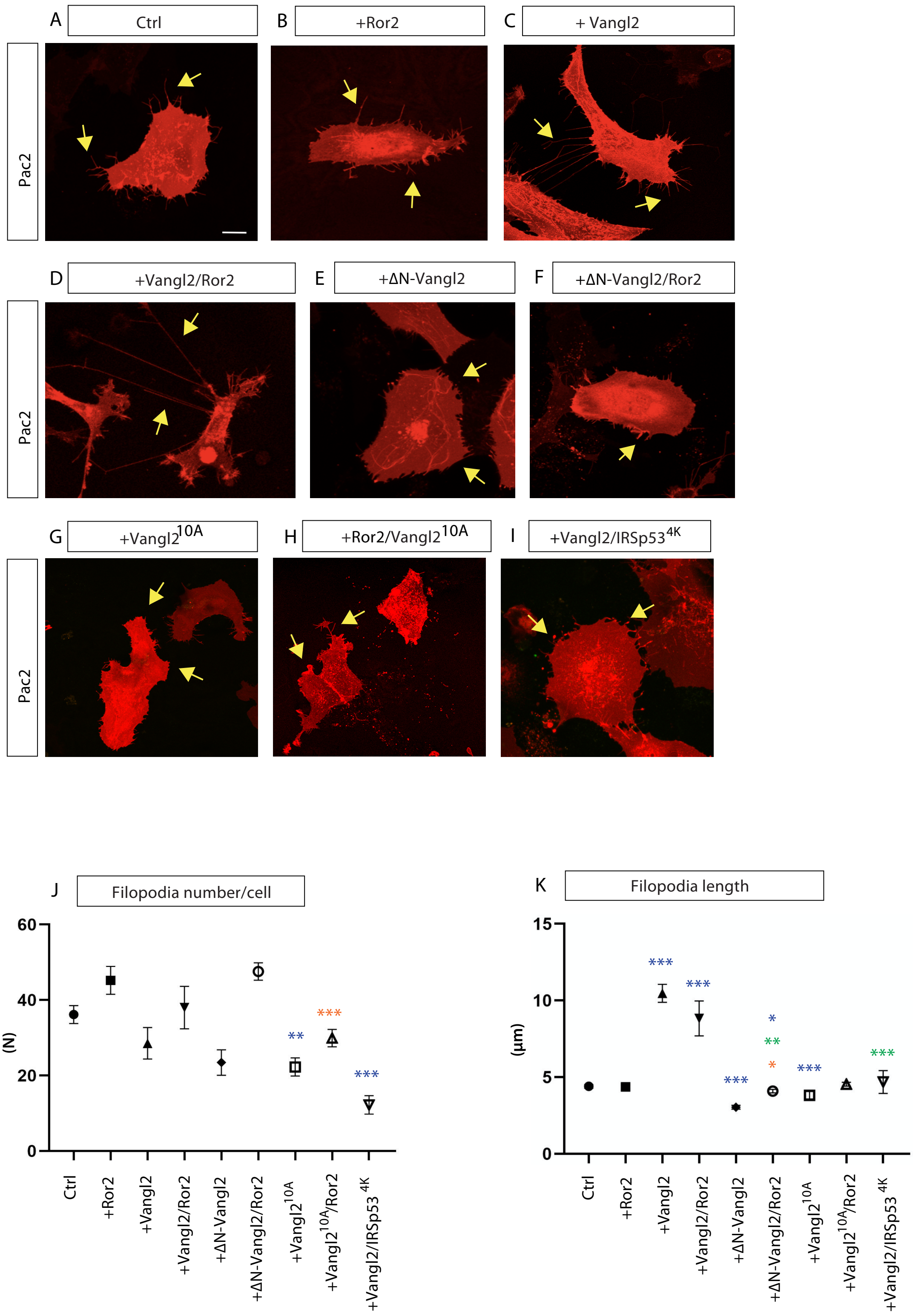

Supplementary Figure 3: Overexpression of Vangl2 in vitro leads to longer filopodia protrusions. (A-I): PAC2 zebrafish fibroblasts transfected with indicated constructs to study filopodia protrusions. Yellow arrows indicate filopodia protrusions. Scale bar= 10µm. (J): Number of filopodia per cell (N = number). (n per condition= 26, 23, 11, 5, 11, 7, 31, 39, 13 cells). (K): Length of filopodia in PAC2 (µm). (n per condition= 975, 1515, 699, 201, 423, 1371, 710, 1245, 159 filopodia). Graphs represent mean and standard error of the mean. Statistical significance: * ≤ 0.05, ** ≤ 0.01, *** ≤ 0.001. Blue stars significant to control, orange stars significant to Ror2, grey stars to Vangl2. One-way ANOVA plus Tukey post-hoc test (J) & two-sided Kruskal-Wallis tests with Bonferroni correction for multiple tests (K). SEM=1. Source data are provided as a Source Data file.

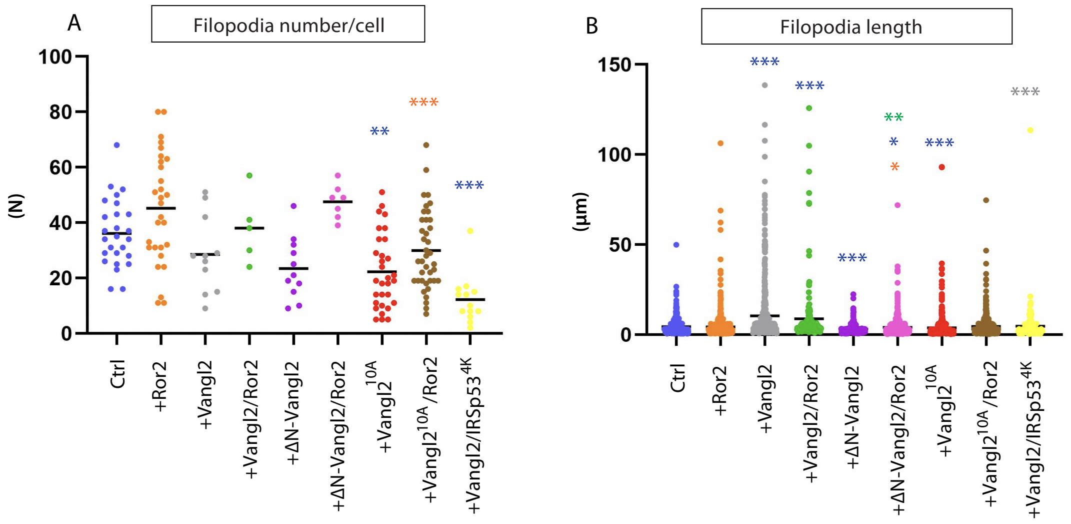

Supplementary Figure 4: Dot plots to show overexpression of Vangl2 in vitro leads longer, more static filopodia. (A-B): Representations of filopodia data from supplementary figure 3 (J,K) as dot plots (A, B) respectively. (A): Number of filopodia per cell (N = number). (n= 26, 29, 11, 5, 11, 7, 31, 39, 13 cells). Stars are significant to Ror2. (B): Length of filopodia in PAC2 (μm). (n= 975, 1515, 699, 201, 423, 1371, 710, 1245, 159 filopodia). Statistical significance: * ≤ 0.05, ** ≤ 0.01, *** ≤ 0.001. Blue stars significant to control, orange stars significant to Ror2, grey stars to Vangl2. One-way ANOVA plus Tukey post-hoc test (A) & two-sided Kruskal-Wallis tests with Bonferroni correction for multiple tests (B). Source data are provided as a Source Data file.

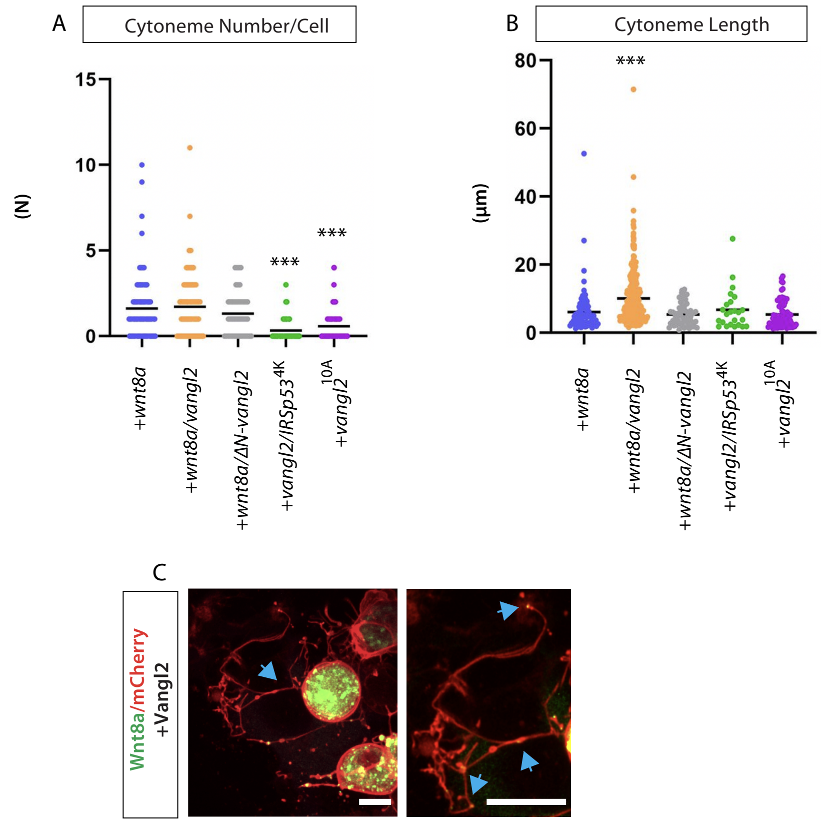

Supplementary Figure 5: Analysis of cytoneme behaviour in zebrafish embryos. (A-B): Representations of number and length of cytonemes in vivo from Figure 3 (H, I) as dot plots (A-B) respectively. (A): Number of Wnt8a positive cytonemes per cell (N = number). (n= 3, 6, 3, 9, 7 embryos, n= 27, 123, 21, 154, 93 cells). (B): Length of Wnt8a positive cytonemes (µm). (n= 81, 269, 54, 82, 24 cytonemes). Statistical significance: * ≤ 0.05, ** ≤ 0.01, *** ≤ 0.001. Two-sided Kruskal-Wallis tests with Bonferroni correction for multiple tests. (C): In vivo Vangl2, Wnt8-GFP and mem-mCh expressing cells exhibiting branched cytonemes with multiple Wnt8a positive contact points (blue arrows). n=6 embryos. Scale bar= 10µm. Source data are provided as a Source Data file.

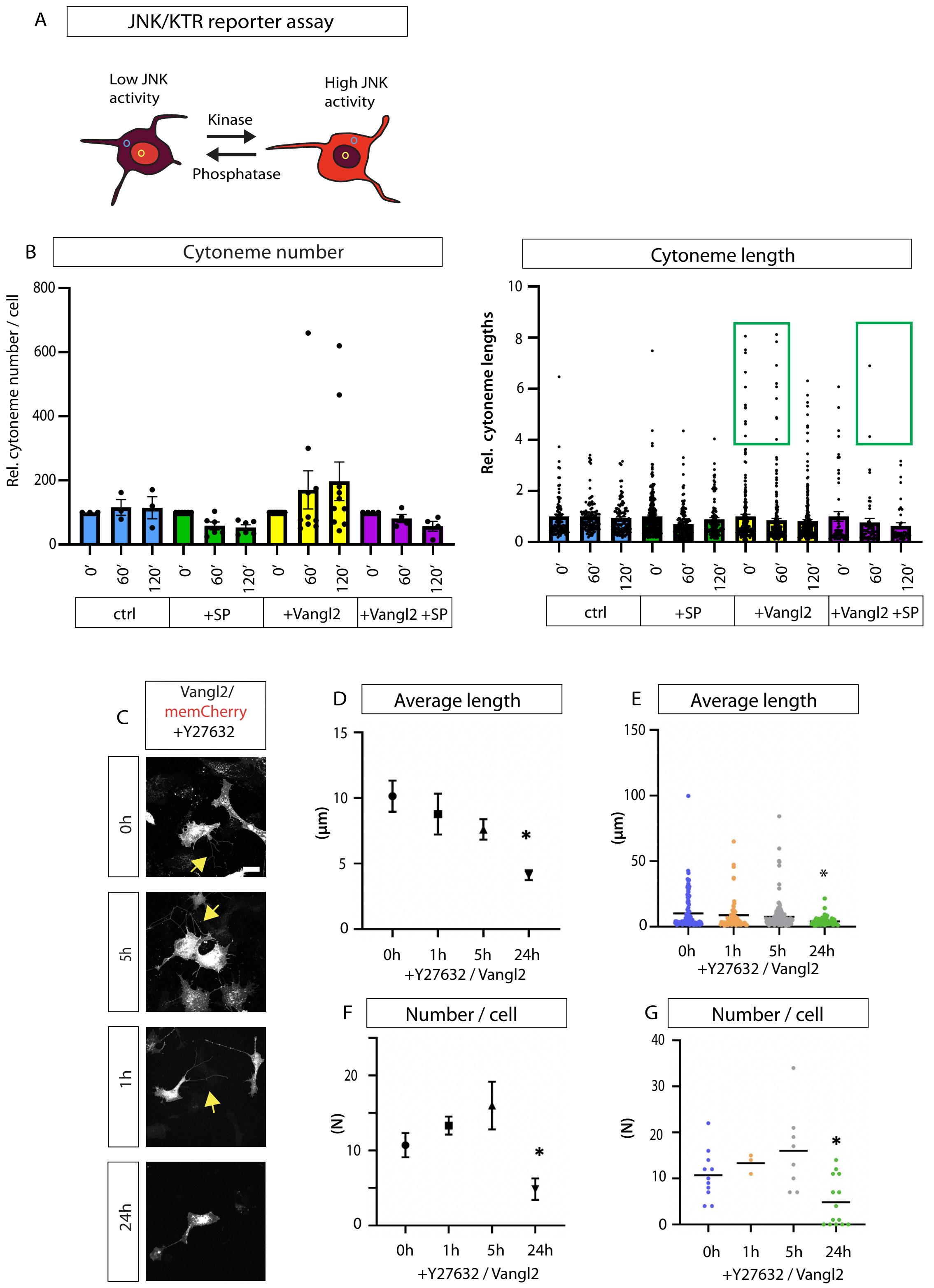

Supplementary Figure 6: JNK and Rock inhibitor treatment on PAC2 fibroblasts. (A): Schematic of KTR-mCherry JNK reporter in HEK293T cells. Low JNK activity in the cell leads to nuclear localisation of KTR-mCherry (yellow circle). High JNK activity leads to phosphorylation and a switch in KTR-mCherry localisation to the cytoplasm and a reduction in nuclear localisation (blue circle). (B): Representation of Figure 4D,E bar charts with dot plots: Relative number of cytonemes per cell in relation to time=0hrs, at 0min, 60min, 120min. (n= 3, 6, 10, 4 cells. n= cytoneme at 0hr, 1hr, 2hr = (102/111/109, 251/156/122, 158/185/215, 58/50/44). Relative cytoneme length (µm) after JNK inhibitor- SP600125 in relation to time=0hrs, at 0min, 60min, 120min. (n= 3, 6, 10, 4 cells. n= cytoneme at 0hr, 1hr, 2hr =

(102/111/109, 251/156/122, 158/185/215, 58/50/44). Green box shows the link between Vangl2/JNK signalling and long cytoneme. (C): PAC2 fibroblasts transfected with GFP-Vangl2 and treated with Rock inhibitor (Y27632) for 1hr, 5hrs and 24hrs. Yellow arrows indicate filopodia. (D-E): Length of filopodia in GFP-Vangl2 expressing cells, 1hr, 5hrs and 24hrs after Rock inhibitor - Y27632 - treatment. (n= 12, 5, 12, 9 cells, n= 121, 63, 171, 68 filopodia). (F-G): Number of filopodia in GFP-Vangl2 expressing cells, 1hr, 5hrs and 24hrs after Rock inhibitor - Y27632 - treatment, (n= 11, 3, 8, 14 cells). Represented as mean and Standard error (D,F) and as dot plots (E,G). Statistical significance: * ≤ 0.05, ** ≤ 0.01, *** ≤ 0.001. Two-sided Kruskal-Wallis tests without Bonferroni correction. SEM=1. Scale bar= 10µm. Source data are provided as a Source Data file.

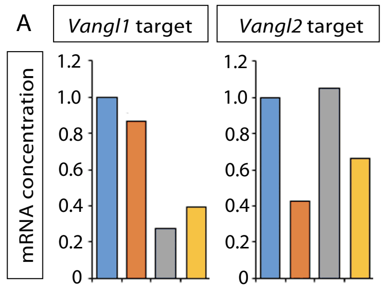

Scrambled siRNA	+	-	-	-		+	-	-	-
Vangl2 siRNA	-	+	-	+		-	+	-	+
*Vangl*1 siRNA	-	-	+	+		-	-	+	+

Supplementary Figure 7: Vangl1 and Vangl2 knockdown in murine telocytes. (A): mRNA concentration of *Vangl1* and *Vangl2* in scrambled siRNA, *Vangl1*, *Vangl2* and *Vangl1/Vangl2* siRNA treated murine telocytes. Primer sequences used for RT-PCR listed in Supplementary Table 1.

Supplementary Table 1: Primer sequences for RT-PCR of *Vangl1* and *Vangl2*.

Primer Name	Primer Sequence
mVangl1 #1 Forward	CGCTTCTACAGCTTGGGACA
mVangl1 #1 Reverse	TGGCTGCTCGGAATTTGGAT
mVangl2 #2 Forward	ACTTCCCTGTCTACAACCCC
mVangl2 #2 Reverse	TTGATTGGCCCGTGGAGTTA
