# Supplementary material for: Vangl2 promotes the formation of long cytonemes to enable distant Wnt/β-catenin signaling
Source: Nat Commun. 2021 Apr 6;12:2058. doi: 10.1038/s41467-021-22393-9 (PMC8024337; doi:10.1038/s41467-021-22393-9)
Supplement: Supplementary file 2 — Description of Additional Supplementary Files [file 41467_2021_22393_MOESM2_ESM.pdf]

Vangl2 promotes the formation of long cytonemes to enable distant Wnt/β-catenin signalling

Lucy Brunt, Gediminas Greicius, Sally Rogers, Benjamin D Evans, David M Virshup, Kyle CA Wedgwood, Steffen Scholpp

Description of additional supplementary information

Title: Supplementary Movie 1A:
Description: 3D rotation of clonal cells in a *Tg(vangl2:GFP-Vangl2)* embryo at 5hpf. Clonal cells express mem-mCherry to show Vangl2-GFP on cytoneme tips. Example A. Scale bar 10µm.

Title: Supplementary Movie 1B:
Description: 3D rotation of clonal cells in a *Tg(vangl2:GFP-Vangl2)* embryo at 5hpf. Clonal cells express mem-mCherry to show Vangl2-GFP on cytoneme tips. Example B. Scale bar 10µm.

Title: Supplementary Movie 2A:
Description: 3D rotation of clonal cells in a *Tg(vangl2:GFP-Vangl2)* embryo at 5hpf. Clonal cells express Ror2-mCherry to show Vangl2-GFP on cytoneme tips. Example A. Scale bar 10µm.

Title: Supplementary Movie 2B:
Description: 3D rotation of clonal cells in a *Tg(vangl2:GFP-Vangl2)* embryo at 5hpf. Clonal cells express Ror2-mCherry to show Vangl2-GFP on cytoneme tips. Example B. Scale bar 10µm.

Title: Supplementary Movie 3:
Description: 3D rotation of clonal cells in a zebrafish embryo at 5hpf. Clonal cells express Wnt8a-GFP and mem-mCherry to show Wnt8a positive cytonemes. Scale bar 10µm.

Title: Supplementary Movie 4:
Description: 3D rotation of clonal cells in a zebrafish embryo at 5hpf. Clonal cells express Wnt8a-GFP and Vangl2 and mem-mCherry to show multiple contact points of Wnt8a positive cytonemes. Scale bar 10µm.

Title: Supplementary Movie 5:
Description: 3D rotation of clonal cells in a zebrafish embryo at 5hpf. Clonal cells express Wnt8a-GFP and $Vangl2^{10A}$ and mem-mCherry to show fewer Wnt8a positive cytonemes. Scale bar 10µm

Title: Supplementary Movie 6:
Description: 3D rotation of clonal cells in a zebrafish embryo at 5hpf. Clonal cells express Wnt8a-GFP and Vangl2 and $IRSp53^{4K}$ and mem-mCherry to show fewer Wnt8a positive cytonemes and ruffled protrusions. Scale bar 10µm

Title: Supplementary Movie 7:
Description: Zebrafish PAC2 cells transfected with Wnt8a-GFP and mem-mCherry. Time stamp and scale bar (30µm) included in movie. 3 frames/second.
