# Supplementary material for: Vangl2 promotes the formation of long cytonemes to enable distant Wnt/β-catenin signaling
Source: Nat Commun. 2021 Apr 6;12:2058. doi: 10.1038/s41467-021-22393-9 (PMC8024337; doi:10.1038/s41467-021-22393-9)
Supplement: Supplementary file 13 — Reporting Summary [file 41467_2021_22393_MOESM13_ESM.pdf]

nature research

Corresponding author(s): Steffen Scholpp

Last updated by author(s): 28/02/2021

Reporting Summary

Nature Research wishes to improve the reproducibility of the work that we publish. This form provides structure for consistency and transparency in reporting. For further information on Nature Research policies, see our Editorial Policies and the Editorial Policy Checklist.

Statistics

For all statistical analyses, confirm that the following items are present in the figure legend, table legend, main text, or Methods section.

n/a	Confirmed	
☐	☒	The exact sample size (*n*) for each experimental group/condition, given as a discrete number and unit of measurement
☐	☒	A statement on whether measurements were taken from distinct samples or whether the same sample was measured repeatedly
☐	☒	The statistical test(s) used AND whether they are one- or two-sided<br>*Only common tests should be described solely by name; describe more complex techniques in the Methods section.*
☐	☒	A description of all covariates tested
☐	☒	A description of any assumptions or corrections, such as tests of normality and adjustment for multiple comparisons
☐	☒	A full description of the statistical parameters including central tendency (e.g. means) or other basic estimates (e.g. regression coefficient) AND variation (e.g. standard deviation) or associated estimates of uncertainty (e.g. confidence intervals)
☐	☒	For null hypothesis testing, the test statistic (e.g. F, t, r) with confidence intervals, effect sizes, degrees of freedom and P value noted<br>*Give P values as exact values whenever suitable.*
☒	☐	For Bayesian analysis, information on the choice of priors and Markov chain Monte Carlo settings
☒	☐	For hierarchical and complex designs, identification of the appropriate level for tests and full reporting of outcomes
☒	☐	Estimates of effect sizes (e.g. Cohen's d, Pearson's r), indicating how they were calculated

Our web collection on statistics for biologists contains articles on many of the points above.

Software and code

Policy information about availability of computer code

Data collection	Image data were visualised and analysed in Leica LAS X software (Version 3.7.2.22383) and FIJI (ImageJ 2.00-rc-59/1.53c). Movies were produced in Imaris 9.0.0 (Bitplane AG).
Data analysis	Statistical analysis carried out using IBM SPSS Statistics 26. Normal multiple comparisons were tested with one-way ANOVA, two-way ANOVA, Students's T-test, Kruskal-Wallis test with Bonferroni correction ,Tukey's post-hoc test as appropriate.

For manuscripts utilizing custom algorithms or software that are central to the research but not yet described in published literature, software must be made available to editors and reviewers. We strongly encourage code deposition in a community repository (e.g. GitHub). See the Nature Research guidelines for submitting code & software for further information.

Data

Policy information about availability of data

All manuscripts must include a data availability statement. This statement should provide the following information, where applicable:

- Accession codes, unique identifiers, or web links for publicly available datasets
- A list of figures that have associated raw data
- A description of any restrictions on data availability

Source data are provided with this paper (Source Data file). The FCCS data that support the findings of this study are available in Dryad, Dataset https://doi.org/10.5061/dryad.cfxpnvx4p. Additional data that support the findings of this study are available from the corresponding author upon reasonable request.

Field-specific reporting

Please select the one below that is the best fit for your research. If you are not sure, read the appropriate sections before making your selection.

☒ Life sciences ☐ Behavioural & social sciences ☐ Ecological, evolutionary & environmental sciences

For a reference copy of the document with all sections, see nature.com/documents/nr-reporting-summary-flat.pdf

Life sciences study design

All studies must disclose on these points even when the disclosure is negative.

Sample size	Sample sizes may be chosen by using a target of power of a statistical test to be applied once the sample is collected.
Data exclusions	No data were excluded from the analyses.
Replication	All experiments were done as three independent experiments. Usually, three biological replicates were analysed. All attempts correctly executed were included.
Randomization	Randomization involved selection of random samples within a experimental /treatment group by the investigators. Usually 10 randomly selected samples (i.e. cells, zebrafish embryos, organoids) were chosen and the parameters analysed. The investigators choosing the samples did not know about the particular treatment.
Blinding	Samples were chosen randomly from a group without giving the investigators access to the treatment protocol.

Reporting for specific materials, systems and methods

We require information from authors about some types of materials, experimental systems and methods used in many studies. Here, indicate whether each material, system or method listed is relevant to your study. If you are not sure if a list item applies to your research, read the appropriate section before selecting a response.

Materials & experimental systems

n/a	Involved in the study	
☐	☒	Antibodies
☐	☒	Eukaryotic cell lines
☒	☐	Palaeontology and archaeology
☐	☒	Animals and other organisms
☒	☐	Human research participants
☒	☐	Clinical data
☒	☐	Dual use research of concern

Methods

n/a	Involved in the study	
☒	☐	ChIP-seq
☒	☐	Flow cytometry
☒	☐	MRI-based neuroimaging

Antibodies

Antibodies used	anti-Wnt8a antibody, MBS9216179, MyBioSource, 1:50; anti-human Vangl2 antibody, SAB2501092, Sigma, 1:50; anti-rabbit antibody Alexafluor 488, ab150077, Abcam, 1:1000; donkey anti-goat antibody AlexaFluor 647, ab150135, Abcam, 1:1000
Validation	All primary antibodies have been validates by IHS and WB as stated in the manuscript.

Eukaryotic cell lines

Policy information about cell lines

Cell line source(s)	PAC2 zebrafish fibroblasts provided by Nicholas Foulkes (KIT, Germany) and Reinhard Koester (TU Braunschweig, Germany) - No commercial source. AGS (Gastric cancer cells) and HEK293T (Human Embryonic Kidney, CRL-1573) - American Tissue Culture Collection, ATTC, Wesel, Germany
Authentication	None of the cell lines were authenticated.
Mycoplasma contamination	All lines were tested for Mycoplasma contamination every three months and confirmed mycoplasma-free.
Commonly misidentified lines (See ICLAC register)	No commonly misidentified cell lines were used in this study.

Animals and other organisms

Policy information about studies involving animals; ARRIVE guidelines recommended for reporting animal research

Laboratory animals	zebrafish, strain AB strain, sex male/female, age 8-18months,
Wild animals	No wild animals were used in the study.
Field-collected samples	No field collected samples were used in the study.
Ethics oversight	WIK wild-type and Tg(vangl2:GFP-Vangl2) 32 zebrafish (Danio rerio) were maintained as previously described at 28°C and on a 14hr light/10hr dark cycle14. Zebrafish care and all experimental procedures were carried out in accordance with the European Communities Council Directive (2010/63/EU) and Animals Scientific Procedures Act (ASPA) 1986. In detail, adult zebrafish for breeding were kept and handled according to the ASPA animal care regulations and all embryo experiments were performed before 120h post fertilization. Zebrafish experimental procedures were carried out under personal and project licenses granted by the UK Home Office under ASPA, and ethically approved by the Animal Welfare and Ethical Review Body at the University of Exeter. All mouse procedures were approved by the Singapore Health Services (SingHealth) Institutional Animal Care and Use Committee.

Note that full information on the approval of the study protocol must also be provided in the manuscript.
